# Triterpenes and Steroids from *Euphorbia denticulata* Lam. With Anti-Herpes Symplex Virus Activity 

**Published:** 2013

**Authors:** Sara Shamsabadipour, Mustafa Ghanadian, Hojjatollah Saeedi, Mohammad Reza Rahimnejad, Marzieh Mohammadi-Kamalabadi, Seyed Majid Ayatollahi, Loghman Salimzadeh

**Affiliations:** a *Isfahan Pharmaceutical Sciences Research Center. Isfahan University of Medical Sciences, Isfahan, Iran. *; b *Department of Pharmacognosy, Isfahan Faculty of Pharmacy and Pharmaceutical Sciences, Isfahan University of Medical Sciences, Isfahan Iran. *; c *Department of Biology, Faculty of Science, University of Isfahan, Isfahan, Iran.*; d *Microbiology, Islamic Azad University, Falavarjan Branch, Young Researchers and Elites Club, Isfahan, Iran. *; e *Phytochemistry Research Center and School of Pharmacy, Shahid Beheshti University of Medical Sciences, Tehran, Iran.*; f *Medical Plants Research Center, Shahrekord University of Medical Sciences, Shahrekord, Iran. *

**Keywords:** *Euphorboa denticulate*, triterpenes, cycloartanes, steroids, toxicity, anti- HSV-1 activity

## Abstract

In this research, dried acetone:chloroform extract of aerial parts of *E. denticulata *as one of the endemic plants to Iran, afforded a number of triterpenes and steroids including: betulin, 24-methylene-cycloart-3-ol, cycloart-23Z-ene-3*β*,25-diol, cycloart-23E-ene-3*β*,25- diol, ergosta-8,24-dien-3-ol (obtusifoliol) and beta-sitosterol which were reported for the first time from this plant. The structure of these compounds was elucidated by NMR and mass spectroscopic methods. The MTS assay was used to determine the toxicity and antiviral activity of betulin and (3*β*,23E)-cycloarta-23-ene-3,25-diol. Betulin showed anti-HSV-1 activity with EC50 value of 84.37±0.02 μg/mL, and toxicity on normal vero cells with CC50 value of 660.718±0.072 μg/mL. (3*β*,23E)-Cycloarta-23-ene-3,25-diol showed antiviral effect with EC50 value of 86.63±0.03 μg/mL, and toxicity with CC50 value of 1089.21±0.25 μg/mL. The results revealed that these two compounds have the antiviral activity far below the CC50 value with selectivity index (CC50/EC50) values of 7.83, and 12.57, respectively.

## Introduction

Euphorbiaceae is one of the largest families of the phylum Anthophyta. In this family the largest genus is *Euphorbia *which comprises well over 2000 species in tropical and temperate zones of Asia and other parts of the world (1). In Iran 70 species are reported that 17 of them are endemic. In traditional medicine *Euphorbia *was used to treat inflammations or as an antivirus or antitumor (2, 3). There are reports on *Euphorbia *species with anti HIV effects or anti herpes simplex virus (3-5). Currently coordinate with the medicinal chemists which rapidly build and develop new synthetic drugs, researchers of the natural product chemistry are also discovering secondary metabolites in plants with their subsequent biological effects. In this context, the paper in hand was also aimed to isolate and detect this type of compounds from *Euphorbia denticulata *Lam., considering that *Euphorbia *genus is one of the rich and economic sources of triterpenoids specially cycloartanes that as intermediates convert to steroids in the plant metabolic pathways (6).

**Figure 1:**
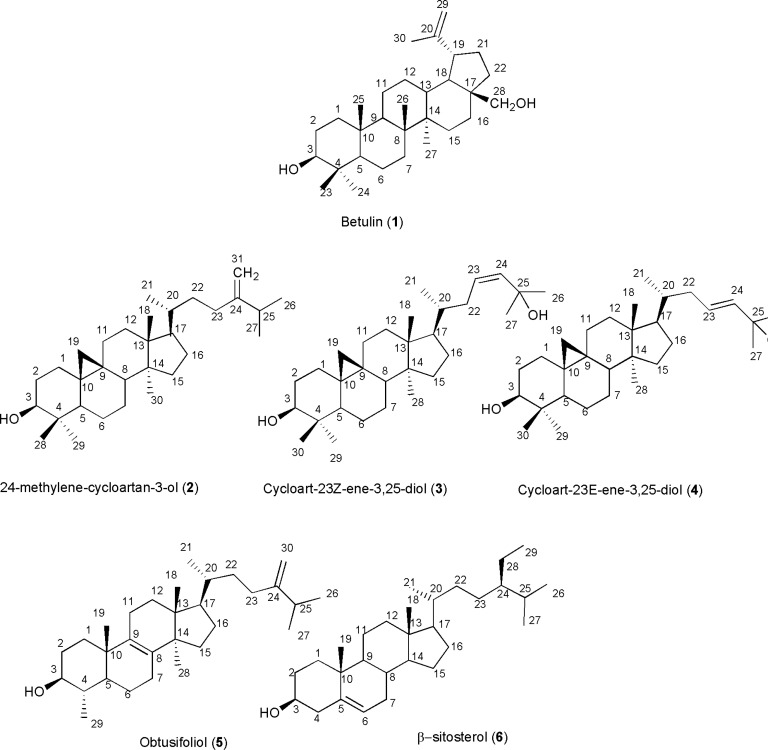
Triterpenes and steroids from *E*uphorbia *denticulate*

## Experimental

*General experimental procedures*

The NMR spectra were recorded on a Bruker Avance AV 400, using CDCl_3_ as solvent. HPLC was carried out on a waters 515 using a Pack-Sil column (250×20 mm i.d.) packed with 5 μm silica (YMC Co., Ltd., Kyoto, Japan) and hexane:EtOAc as mobile phase. Chromatographic materials were silica gel (Merck Co., Germany). Thin layer chromatography detection was achieved by spraying the silica gel plates with cerium sulfate in 10% aq. H_2_SO_4_, followed by heating.

*Materials*

Dulbeccoʼs modified eagleʼs growth medium (DMEM), and Aciclovir purchased from Sigma-aldrich company (St Louis, MO, USA), fetal bovine serum (FBS ), streptomycin, and amphotericin B from the GIBCO/Invitrogen (Karlsruhe, Germany), and MTS [3-(4,5-dimethylthiazol-2-yl)-5-(3-arboxymethoxyphenyl) -2-(4-sulfophenyl) 2H-tetrazolium] from Promega (Madison, WI, USA). African green monkey kidney cell line (Vero cell line, ATCC C102) was obtained from cell repository of tissue culture department, Pasture Institue, Iran. Herpes simplex virus type 1 (HSV-1, strain KOS) obtained from Virology Department of Tarbiat Modares University (Tehran, Iran). 

*Plant material*

Plant material was collected from populations growing in Sanandaj (Iran) at the West part of Iran and identified by Dr. Hojatollah Saeedi in the Department of Biology, Faculty of Science, University of Isfahan and a voucher specimen (#19001) is preserved in the herbarium of the University of Isfahan (Iran). 

*Extraction and isolation*

**Figure 2:**
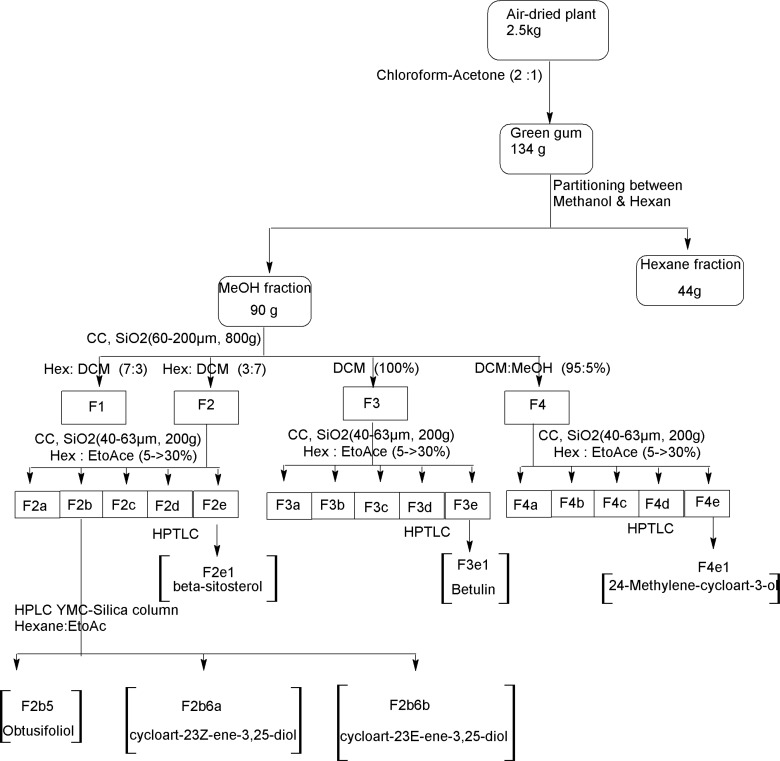
Flowchart representative of extraction and purification processes of triterpenes and steroids from *Euphorbia denticulata*

The air-dried plant material (2.5 kg) was macerated with dichloromethane/acetone 2:1 (20L×3) at room temperature for 5 days. Filtration and *in-vacu*o concentration resulted in a green gum (134 g) which was partitioned between methanol and n-hexane. The defatted methanolic extract was concentrated (90 g) and subjected on silica gel CC (60-200 μm, 800 g) eluting with hexane/ dichloromethane, 0**→**100 to give four fractions: Fr.1-Fr.4. Inferred from TLC and 1H-NMR, Fr.1 (21.3 g) contained alkanes and fats, Fr.2 (15.2 g) containing steroids, and Fr.3 (12.6 g) as well as Fr.4 (18.1 g) triterpenes. Fr.2, Fr.3, and Fr.4 were chromatographed on flash silica gel (40-63 μm, 200 g) using hexane/ethyl acetate, 5**→**30. Finally steroids and triterpenes were further purified on preparative layer chromatography or high pressure liquid chromatography (HPLC) with YMC-Pak-Sil column (250 × 20 mm) and hexane:ethyl acetate (80:20) as mobile phase to yield compounds 1-6 (Figure 2).

*Betulin: lup-20(29)-ene-3β,28-diol (1)*

White crystals; MW(g/mol): 442; yield: 0.01%; 1H-NMR (CDCl_3_, 400 MHz): δH 4.61 (^1^H, d, *J= *2.4 Hz, H-29a), 4.51 (^1^H, dd, *J= *2.4, 1.6 Hz, H-29b), 3.73 (^1^H,dd, *J= *10.8, 1.6 Hz, H-28a), 3.26 (^1^H, br d, *J= *10.8, Hz, H-28b), 3.12 (^1^H, dd, *J= *11.6, 5.2 Hz, H-3), 2.32 (^1^H, dt, *J= *10.8, 6.0 Hz, H-19), 1.61 (3H, s, H-30), 0.95 (3H, s, H-26), 0.91 (3H, s, H-23), 0.90 (3H, s, H-27), 0.75 (3H, s, H-25), 69 (3H, s, H-24); 13C-NMR data: see Table 1. EIMS *m/z *442 [M], 411, 234, 220, 207, 203, 189, 175, 165, 135, 105, 67, 55, 41.

**Table 1:** 13C-NMR chemical shifts of the triterpenes and steroids in *Euphorbia denticulata*

**C**	**1**	**2**	**3**	**4**	**5**	**6**	**C**	**1**	**2**	**3**	**4**	**5**	**6**	
**1**	38.7	32.0	32.0	32.0	35.0	37.3	**16**	29.2	26.5	26.4	26.4	31.0	28.2	
**2**	25.2	30.4	30.4	30.4	31.1	31.7	**17**	47.8	52.3	52.1	52.1	50.4	56.8	
**3**	79.0	78.8	78.8	78.8	76.5	71.8	**18**	47.8	18.1	18.1	18.1	15.7	12.2	
**4**	38.9	40.5	40.1	40.5	39.2	42.3	**19**	48.7	29.9	29.9	29.9	18.7	19.4	
**5**	55.3	47.1	47.1	47.1	47.0	140.8	**20**	150.5	36.4	36.3	36.3	36.5	36.1	
**6**	18.3	21.1	21.1	21.1	20.7	121.7	**21**	29.7	18.2	18.4	18.4	18.2	18.8	
**7**	34.2	28.0	28.1	28.1	28.2	31.9	**22**	34.0	35.6	39.4	39.4	35.0	34.0	
**8**	40.9	48.0	48.0	48.0	133.6	31.9	**23**	28.0	31.3	130.9	130.9	30.8	26.1	
**9**	50.4	20.0	20.0	20.0	134.6	50.2	**24**	15.4	157.0	134.4	134.4	156.9	45.9	
**10**	37.2	25.8	26.1	26.1	36.4	36.5	**25**	16.0	33.8	70.9	70.9	33.8	29.2	
**11**	20.8	26.0	26.0	26.0	21.8	21.1	**26**	16.1	22.0	24.4	24.4	21.9	19.8	
**12**	25.2	32.9	32.8	32.8	25.5	39.8	**27**	14.8	19.3	24.3	24.3	22.0	19.1	
**13**	37.3	45.3	45.5	45.3	44.6	42.3	**28**	60.6	18.3	19.3	19.3	15.1	231	
**14**	42.7	48.8	47.9	48.8	49.9	56.1	**29**	109.7	14.0	14.0	14.0	105.9	12.1	
**15**	27.0	35.0	35.6	35.6	31.3	24.3	**30**	19.1	25.5	25.4	25.4	24.4		

*24-methylene-cycloart-3β-ol (2)*

White crystals; MW(g/mol): 426; yield: 0.001%; 1H-NMR (CDCl_3_, 400 MHz): δH 4.69 and 4.64 (each ^1^H, br s, H-31a,b), 3.25 (^1^H, dd, *J=*11.0 , 4.0 Hz, H-3), 1.01 (3H, d, *J=*3 Hz, H-27), 0.99 (3H, d, *J=*3 Hz, H-26), 0.94 (3H, s, 6H: H-18, H-30 ), 0.88 (3H, s, H-28), 0.86 (3H, d, *J=*6 Hz, H-21) and 0.79 (3H, s, H-29), 0.53 and 0.31 (each ^1^H, d, *J= *4.5 Hz, H-19a,b); 13C-NMR data: see Table 1; EIMS *m/z*: 426, 379, 365, 353, 300, 286, 203, 175, 161, 147, 121, 107, 95, 81, 69, 55, 41. 

*Cycloart-23Z-ene-3β,25-diol (3)*

White crystals; MW(g/mol): 442; yield: 0.00024%; 1H-NMR (CDCl_3_, 400 MHz): δH 5.62 (2H, br s, H-23,24), 3.30 (^1^H, dd, *J=*4.0, 11.2 Hz, H-3), 1.33 (6H, s, H-26, H-27), 0.99 (6H, s, H-18, H-30), 0.90 (3H, s, H-28), 0.89 (3H, d, *J=*6.4 Hz, H-21), 0.83 (3H, s, H-29), 0.57, 0.35 (each ^1^H, d, *J=*4.4 Hz, H-19a, b) ); 13C-NMR data: see Table 1; EIMS *m/z*: 442 [M]^+^, 427, 424, 381, 363, 355, 315, 313, 302, 269, 175.

*Cycloart-23E-ene-3β,25-diol (4)*

White crystals; MW(g/mol): 442; yield: 0.0002%; ^1^H-NMR (CDCl_3_, 400 MHz): δH 5.72 (1H, m, H-23), 5.54 (^1^H, d, *J=*15.6 Hz, H-24), 3.29 (^1^H, m, H-3), 1.59 (3H, s, H-26), 1.37 (3H, s, H-27), 1.0 (3H, s, H-29), 0.99 (3H, s, H-18), 0.90 (3H, s, H-30), 0.88 (3H, d, *J= *6.4 Hz, H-21), 0.83 (3H, s, H-28), 0.57 and 0.35 (each ^1^H, d, *J=*4.4 Hz, H-19a, b)); 13C-NMR data: see Table 1. EIMS *m/z*: 442, 424, 409, 407, 315, 302, 297, 255, 203, 187, 175, 145, 43.

*Obtusifoliol: *4,14-dimethyl ergosta-8,24(28)-*dien-3β-ol (5)*

White crystals; MW(g/mol): 426; yield: 0.00044%; 1H-NMR (CDCl3, 400 MHz): δH 4.56 and 4.51 (each ^1^H, br s, H-30a,b), 2.96 (1H, ddd, *J= *4.8, 10.0, 14.8 Hz, H-3), 0.89 (3H, d, *J= *6.8 Hz, H-27), 0.88 (3H, d, *J= *6.8 Hz, H-26), 0.85 (3H, d, *J= *6.4 Hz, H-21), 0.82 (3H, s, H-19), 0.79 (3H, d, *J= *6.4, , H-29), 0.74 (3H, s, H-28), 0.55 (3H, s, H-18); 13C-NMR data: see Table 1; EIMS *m/z*: 426, 393, 327, 259, 245, 233, 173, 159, 69. 

*β- sitosterol: Stigmast-5-en-3β -ol (6)*

White crystals; MW(g/mol): 414; yield: 0.0013%; ^1^H-NMR (CDCl_3_, 400 MHz): δH 5.38 (^1^H, br d, *J= *5.2 Hz, H-6), 3.54 (^1^H, m, H-3), 1.03 (3H, s, H-19), ), 0.95 (3H, d, *J= *6.4 Hz, H-21), 0.88 (3H, d, *J= *7.6 Hz, H-24) , 0.86 (3H, d, *J= *6.8 Hz, H-27), 0.84 (3H, d, *J= *6.8 Hz, H-26), 0.69 (3H, s, H-18).; 13C-NMR data: see Table 1; EIMS *m/z*: 414, 329, 303, 273, 255, 231, 213, 161, 119, 107, 105, 95, 79), 71, 69, 67, 57, 55.

*Virus and cells*

For cytotoxicity and antiviral assays cells were grown in Dulbeccoʼs Modified Eagleʼs growth Medium ( DMEM ; Sigma, USA) supplemented with 2% of fetal bovine serum (FBS; Gibco, Germany), 100 μg/mL of streptomycin, 100 UI/mL of penicillin and 0.25 μg/mL amphotericin B ( Gibco, Germany) and 0.14% (v/v) sodium bicarbonate. All the cells were cultured at 37°C in a humidified atmosphere supplied with 5% CO2.

Stock preparations of the herpes simplex virus type 1 (HSV-1, strain KOS), were generated by incubating in Vero cells (75 cm^2^ culture flasks seeded with 350000 cells/mL). After 72 h infection, the cultures were frozen and thawed twice before centrifugation and the resulting supernatant aliquots stored at -70°C. Virus titers were determined by cytopathic effects in Vero cells and were expressed as 50% Tissue Culture Infective Dose (TCID50) per mL (7, 8). *Cytotoxicity assay *

The Vero cells were seeded onto a 96- well plate at a concentration of 3.5×10^5^ Vero cells per mL and a volume of 100 μL per well. Following 24 h incubation at 37 °C, a confluent cell monolayer was confirmed and cell media was removed. Test compounds were serially diluted with the culture medium supplemented with 2% serum to reach the different concentrations. Negative control dilution of DMSO at 0.1 % was also included. An aliquot of 100 μL/well of each diluted compound or DMSO was added to the plates in triplicate. After incubation at 37 °C with 5% CO_2_ for 3 days, MTS (Cell Titer 96; Promega, USA) was added to each well with a volume of 20 μL. The trays were further incubated for 2 h to allow MTS production. The absorbances were determined with an ELISA reader (Stata Fax 2100, USA) at a test wavelength of 490 nm. Data were calculated as the percentage of inhibition using the following formula: inhibition % =[100 – ( At/ As) × 100]%. At and As refer to the absorbances of the test substances and the solvent control, respectively. CC50 values, defined as the concentration of 50% cellular cytotoxicity (CC50) of test compounds (7, 8). 

*Antiviral assay using MTS method*

The antiviral activity of compounds isolated from *E. denticulata *against HSV-1 were evaluated by the MTS method. Vero cells, treated with trypsin, were seeded onto 96-well plates with a concentration of 5 × 10^3^ cells in a volume of 100 μL per well. After incubation at 37 °C with 5% CO_2_ for 24 h, when the cell monolayers were confluent, the medium was removed from the wells and 100 μL of test virus was added and incubated for another 2 h. Different non-cytotoxic concentrations ( ≤ CC50 values) of test compounds were then added to culture wells in triplicate. The maximum concentration of DMSO (0.1%) was used as a negative control. Aciclovir; Sigma, USA) was used as a positive control for HSV-1. After incubation at 37°C with 5% CO_2_ for 3 days, the MTS test was carried out as previously described. The percentages of protection were calculated as [(A − B) × 100/( C−B)], where A, B, and C indicate the absorbances of test compounds, virus and cell controls, respectively. Each obtained EC50 value was defined as the effective concentration that reduced the absorbance of infected cells to 50% when compared with cell and virus controls (7, 9).

*Time- course anti-virus antanalysis of isolated compounds*

Different non-cytotoxic concentrations (≤ CC50 values) of test compounds were added to culture cells in triplicate at different times pre-infection or post-infection. HSV-1(10 TCID50 per well) was inoculated onto confluent monolayers of Vero cells for 2 h. After 3 days, MTS test and antiviral activity were carried out as previously described (3).

*Statistical analysis*

The selectivity index (SI) was determined as the ratio of CC50 to EC50.The statistically different effects of test compounds on the inhibition of HSV -1 were compared with the control group or together using the Student ’s t-test. 

## Result and Discussion

Compound 1, was identified as C_30_H_50_O_2_ on the basis of EI-MS of *m/z *442, and 13C-NMR (BB & DEPT) spectral data indicating six methyls, twelve methylenes, six methines, and six quaternary carbons which two of them were oxygenated. 1H-NMR revealed six singlet methyls at *δH *1.61 (H-30), 1.18 (s, H-27), 0.95 (H-26), 0.91 (H-23), 0.90 (H-27), 0.75 (H-25), and 69 (H-24) along with a pair of olefinic protons as part of an exocyclic-methylene group at *δ*H 4.66 and 4.56 characteristic for lupane triterpenes (10). Two geminal oxymethylene was also detected at *δ*H 3.77 and 3.31 with coupling constant of 10.8 Hz, and one doublet of doublet oxymethine proton at *δ*H 3.12 indicating of 3*β*-hydroxyl group. Taken together, and confirmed from the literature the structure of compound 1 detected as betulin (10, 11). The resonances of compound 2 with EI-MS *m/z *426, encompassed thirty-one carbons including seven methyls, twelve methylenes, six methines and six quaternary carbons. ^1^H-NMR revealed four singlet methyls, a pair of doublets *δ*H 0.53 and 0.31 in the upfield area indicative of cyclopropane ring characteristic of cycloartanes. A doublet of doublet proton at δH 3.25 (dd, *Jax,ax=*11.0 , Jax,eq=4.0 Hz, H3), was indicative of 3*β *-hydroxyl group, and one pair of olefinic protons *δ*H 4.64 and 4.69 (each ^1^H, br s) related to exocyclic terminal methylene. Therfore, comparing to the literature compound 2 determined as 24-methylene-cycloart-3-ol (12). 1H-NMR of compound 3 showed two downfield singlet methyls at δH 1.33 (6H, H-26, H-27) together with four other singlet methyls at *δ*H 0.99 (6H, H-18, H-30), 0.90 (H-28), 0.83 (H-29), and one secondary methyl *δ*H 0.89 (d, *J=*6.4 Hz, H-21) along with a pair of doublets in the upfield area with constant coupling of 4.4 Hz at *δ*H 0.57, 0.35 ppm, characteristic of cycloartane cyclopropane ring. Carbinolic proton at *δ*H 3.30 was related to 3-hydroxyl group and two vicinal olefinic protons overlapping on each other at *δ*H 5.62 (1H, m, H-23), and 5.62 (1H, brs, H-24), with low coupling constants indicative of the *cis *geometry. Therefore based on EI- MS *m/z *442 and consistency of 13C- and 1H-NMR with other reported data in the literature,, compound 3 was identified as cycloart-23Z-ene-3*β*,25-diol (11). Compound 4 with EI-MS of *m/z *442 was also identified as cycloart-23E-ene-3*β*,25-diol based on its similarities with compound 3 except for vicinal olefinic protons at *δH *5.72 (^1^H, m, H-23), and 5.54 (^1^H, d, *J=*15.6 Hz, H-24), with large coupling constant which was indicative of *trans *Δ23-geometry (12,13). The resonances of compound 5, with EI-MS of *m/z *426, encompassed thirty carbons including seven methyls, ten methylenes, seven methines, and six quaternary carbons. ^1^H-NMR revealed three singlet methyls, *δH *0.82 (H-19), 0.74 (H-28), and 0.55 (H-18) with a doublet of doublet of doublet proton at *δH *2.96 (ddd, *J= *4.8, 10.0, 14.8 Hz), indicative of the hydroxyl group at C-3 of 4*α*-methyl steroids, along with one pair of olefinic protons *δH *4.51 (br-s) and 4.56 (br-s) related to exocyclic terminal methylene which were in good agreement with obtusifoliol (14). Compound 6, was also determined as *β*-sitosterol based on EIMS *m/z *414, and 1H-NMR comprised of two singlet methyls *δ*H 1.03 (H-19), and 0.69 (H-18), four secondary methyls at *δ*H 0.95 (d, *J= *6.4 Hz, H-21), 0.88 (d, *J= *7.6 Hz, H-24) , 0.86 (d, *J= *6.8 Hz, H-27), and 0.84 (d, *J= *6.8 Hz, H-26), with one multiplete oxymethine proton at *δH *3.54 indicative of 3-hydroxy group and one broad doublet olefinic proton at *δ*H 5.38 (br d, *J= *5.2 Hz, H-6) (15).

*Assessment of cytotoxicity and anti-HSV-1 activity by MTS assay on Vero cell*

The MTS assay was used to determine the toxicity and antiviral activity of the tested agents (9, 16). Betulin and (3 *β*,23E)-Cycloarta-23-ene-3,25-diol isolated from *E.denticulata *were investigated for anti-HSV-1 activity, were firstly tested for their cytotoxic effect alone on Vero cells. The results revealed that both compounds have antiviral activity far below the CC50 doses (Table 2). Results presented in Table 2 revealed that the CC50 of betulin and (3*β*,23E)-Cycloarta-23-ene-3,25-diol were 660.718±0.072 and 1089.205±0.250 μg/mL, respectively. The results also showed that the rate of cells death increased with increasing the concentration of the tested compounds. 

After studying the cytotoxicity effect of samples on normal Vero cells, the experiments to assess the antiviral effect of samples on HSV-1 were done. 

The anti-HV-1 activity tested by MTS assay showed that betulin and (3*β*,23E)-Cycloarta-23-ene-3,25-diol had anti-HSV-1 activity at different dose levels, based on their EC50 value and selectivity index (SI). In comparison with acyclovir as a standard drug with the SI value of 42.66, the SI value of (3*β*,23E)-Cycloarta-23-ene-3,25-diol was 12.57 and betulin was 7.831 (Table 2). 

**Table 2:** Cytotoxicity and anti-HSV-1 activity of compounds isolated from *E.denticulata*

**Test compounds**	**CC50** **a** **±SEM**	**HSV-1 **	
		**EC50** **b** **±SEM **	**SI** **c **
Betulin	660.72±0.072	84.37±0.018	7.83
*β*,23E)-Cycloarta-23-ene-3,25-diol 3(	1±0.2501089.2	86.63±0.03	12.57
Acyclovir	128.00±0.215	±0.001 3.00	42.66

^a^The 50% cytotoxic concentration on vero cells in μg/mL (n=3).

^b^Concentration of compound (μg/mL) producing 50% inhibition of virus incluced cytopathic effects of three seprate experiments. cSelectivity index (SI) = CC50/EC50

*Time- course anti-virus analysis of isolated compounds*

**Figure 3:**
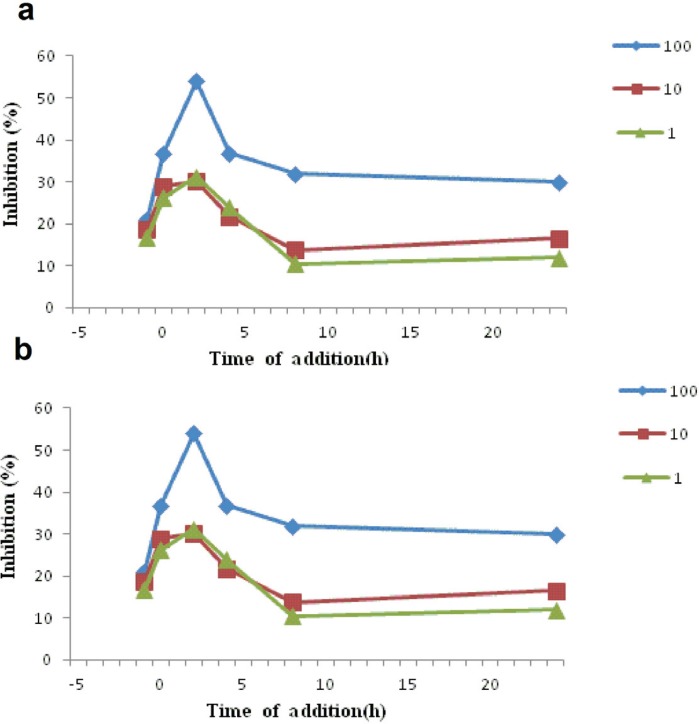
Inhibitory effect of test compounds at different concentrations (1, 10, 100 μg/mL) at various times pre-infection , co-infection and post-infection of herpesvirus (HSV-1) to Vero cells. a) betulin, and b) (3*β*,23E)-Cycloarta-23-ene-3,25-diol were added with the HSV-1 infected Vero cells at various times period like pre-infection (− 1 h), co-infection (0 h) or post-infection( 2-24 h). After 3 days of incubation at 37°C, inhibition was evaluated by MTS assay and expressed as the inhibition percentage. Each point represents the mean of three independent experiments

In order to investigate the mechanism of how each compound inhibits the infection of HSV-1 a study was conducted to investigate the time-course effect at 1 h before to 24 h after the virus infection and using different concentrations of each compound (1, 10, 100 μg/mL). Inhibition was evaluated by MTS assay after 3 days of infection and expressed as percentage inhibition. As it is clear from Figure 3a and Figure 3b, the both compounds betulin and (3*β*,23E)-Cycloarta-23-ene-3,25-diol, exhibited the highest inhibition against HSV-1 infection within 2.0 h post-infection which were during the early period of virus replication.

## Conclusion

*E. denticulata *as one of the endemic plants to Iran, could be a new source of 4,4 dimethyl steroids like 24-methylene-cycloart-3-ol, cycloart-23Z-ene-3*β*,25-diol, and cycloart-23E-ene-3*β*,25-diol as well as obtusifoliol as 4*α*-methyl steroid and beta-sitosterol as 4-desmethyl steroid which were reported for the first time from this plant. In addition *E. denticulata *could be considered as one on the economic sources of betulin (0.01 % of dry weight of the plant). 

Betulin and (3*β*,23E)-Cycloarta-23-ene-3,25-diol showed EC50 value of 84.37±0.02, and 86.63±0.03 μg/mL, and selectivity index (CC50/EC50) values of 7.83, and 12.57, respectively. It shows that both compounds assert their antiviral activity with ignorable toxicity on vero cells.
